# Subgrouping the Autism “Spectrum": Reflections on DSM-5

**DOI:** 10.1371/journal.pbio.1001544

**Published:** 2013-04-23

**Authors:** Meng-Chuan Lai, Michael V. Lombardo, Bhismadev Chakrabarti, Simon Baron-Cohen

**Affiliations:** 1 Autism Research Centre, Department of Psychiatry, University of Cambridge, Cambridge, United Kingdom; 2 Department of Psychiatry, National Taiwan University Hospital, Taipei, Taiwan; 3 School of Psychology and Clinical Language Sciences, Centre for Integrative Neuroscience and Neurodynamics, University of Reading, Reading, United Kingdom; Mount Sinai School of Medicine, United States of America

## Abstract

DSM-5 has moved autism from the level of subgroups (“apples and oranges") to the prototypical level (“fruit"). But making progress in research, and ultimately improving clinical practice, will require identifying subgroups within the autism spectrum.

The biology of autism cannot yet be used diagnostically, and so—like most psychiatric conditions—autism is defined by behavior [Rett syndrome (Rett's disorder) is diagnosed by incorporating biology, but it has been moved out of the “Autism Spectrum Disorder" category in DSM-5]. The two international psychiatric classification systems (the Diagnostic and Statistical Manual of Mental Disorders [DSM] and the International Classification of Diseases [ICD]) remain useful for making clinical diagnoses, but each time these classification systems are revised, the new definitions inevitably subtly change the nature of how the conditions are construed. While acknowledging concerns about issues such as diagnostic inflation [1] and financial conflicts of interest [2], DSM-5 is now “set in stone" and will be published in May 2013. Although this manual is primarily designed for creating a common language for clinical practice, it is also often used in research settings to define the conditions to be studied. Here we reflect on what the revision may mean for research, and for understanding the nature of autism.

New in DSM-5 is the explicit recognition of the “spectrum" nature of autism, subsuming and replacing the DSM-IV Pervasive Developmental Disorder (PDD) categorical subgroups of “autistic disorder," “Asperger's disorder," “pervasive developmental disorder not otherwise specified," and “childhood disintegrative disorder" into a single umbrella term “Autism Spectrum Disorder" (ASD). [Here and throughout we use the term “ASD" because this is what is used in DSM-5. However, in our publications over many years we have opted for the more neutral term “ASC" (Autism Spectrum Conditions) to signal that this is a biomedical diagnosis in which the individual needs support, and which leaves room for areas of strength as well as difficulty, without the somewhat negative overtones of the term “disorder," which implies something is “broken."] DSM-5 characterizes ASD in two behavioral domains (difficulties in social communication and social interaction, and unusually restricted, repetitive behaviors and interests) and is accompanied by a severity scale to capture the “spectrum" nature of ASD.

Also new in DSM-5, language development/level is treated as separate from ASD. This means an individual can have ASD *with* or *without* a language disorder. Finally, DSM-5 proposes a more inclusive age-of-onset criterion, recognizing that although symptoms should present in early childhood, they may not fully manifest until social demands exceed the capacity of the individual to cope with them. The major rationale behind these changes is to improve reliability [3]. The DSM-5 field trial in North America has shown that ASD diagnosis has reasonable test-retest reliability, with an intraclass Kappa (a statistical measure of reliability) of 0.69 (95% CI 0.58–0.79) [4].

There have been concerns that the DSM-5 criteria may be more stringent than DSM-IV, such that some individuals who qualified for PDD will not meet the new ASD criteria. A series of studies testing the initial [5] and revised draft ASD criteria [6]–[12] showed increased specificity but decreased sensitivity of the DSM-5 draft compared to DSM-IV, and suggested relaxation of the threshold (e.g., fewer numbers of required symptom subdomains) to achieve reasonable sensitivity. However, most of these studies suffer from the weakness of using retrospective datasets and tools developed earlier that may not satisfactorily capture symptoms now included in DSM-5 [13]. One prospective study tested both DSM-IV and DSM-5 criteria against the gold standard of “best-estimate clinical diagnoses" and agreed with the “too-stringent" conclusion in a clinical sample [14]. However, another substantially large retrospective study using data from three existing datasets found few differences between the two systems in sensitivity [15].

In brief, these studies all show that DSM-5 provides better specificity (so reducing false-positive diagnoses), but at the expense of potentially reduced sensitivity, especially for older children, adolescents and adults, individuals without intellectual disability, and individuals who previously met criteria for diagnoses of DSM-IV “Asperger's disorder" or “pervasive developmental disorder not otherwise specified." It remains to be seen in real-life settings how diagnostic practice, service delivery, and prevalence estimates will be affected by applying DSM-5 ASD criteria. In particular, one major nosological issue is to what extent individuals fitting DSM-IV PDD but not DSM-5 ASD diagnoses will end up falling into the newly created diagnosis of “Social (Pragmatic) Communication Disorder" [12],[16]–[18]. Clearly more research needs to be done to provide a thorough and fair evaluation of this revision.

Highlighting the dimensional nature of the two cardinal behavioral domains of ASD, as well as the improved organization of symptom descriptions, are excellent features of DSM-5. A unitary label of “ASD" accompanied by individualized assessment of needs for support will likely be useful in clinical settings, especially to guarantee the required levels of support for all individuals “on the spectrum" who will benefit from educational, occupational, social, mental health, and medical interventions (even if they are etiologically, developmentally, and clinically heterogeneous). However, this approach is not useful for research in general, given the known massive heterogeneity within such an omnibus label. Within autism there is a huge variability in terms of behavior (symptom severity and combination), cognition (the range of deficits and assets), and biological mechanisms. Acknowledging heterogeneity has led to the idea that there are many “autisms," with partially distinct etiologies, nested within the umbrella term of “ASD" [19]. Therefore, two critical issues need to be addressed: a clarification of the meaning(s) of the term “spectrum"; and the need for subgrouping.

## What Do We Mean by the Autism “Spectrum"?

There are several meanings of the term “spectrum" in relation to autism. The differences are subtle but nontrivial. DSM-5 does not tease these apart, but in relation to future research into the “autism spectrum," it is important to be clear to which meaning the term “spectrum" refers.

1. “Spectrum" can refer to the dimensional nature of the cardinal features of autism *within the clinical population* (i.e., differences in the severity and presentation of symptoms among those with a diagnosis of ASD). This was suggested in the 1970s, before autism appeared in DSM-III, when Lorna Wing highlighted the diversity among the cardinal behavioral domains within autism [20].
2. “Spectrum" can also refer to the *continuity between the general population and the clinical population*. This view of the spectrum requires the concept of “autistic traits" (sometimes referred to as “autistic-like traits") that run right through the whole population. Autistic traits can refer to the individual features that together comprise the quantitative variability in the *cardinal* behavioral domains defined by DSM/ICD criteria. Studies using questionnaire measures of autistic traits (e.g., the Quantitative Checklist for Autism in Toddlers [Q-CHAT] [21], the Childhood Autism Spectrum Test [CAST] [22], and the Autism Spectrum Screening Questionnaire [ASSQ] [23]) show a continuous distribution of scores, supporting the concept of a spectrum extending into the general population. Underlying autistic traits are genetic susceptibilities that are common across the general population and at the extreme ends [24], and for clinically defined ASD [25]. “Autistic traits" can also refer to *associated features* not described in DSM/ICD criteria, exemplified by items within questionnaires such as the Social Responsiveness Scale (SRS) [26] (e.g., “Becomes upset in a situation with lots of things going on") or the Autism Spectrum Quotient (AQ) [27] (e.g., “I tend to notice details that others do not"). These composite measures of autistic traits (including *both* cardinal and associated features) are also continuously (and normally) distributed, and have been used to characterize the “broader autism phenotype" (BAP) [28],[29]. These traits also show shared genetic association with clinically defined ASD [30].
3. “Spectrum" can also refer to *subgroups* [31],[32]. It has been suggested that “the autisms" may be a useful concept to reflect the substantial heterogeneity within the autistic spectrum [19]. DSM-5 has tried to move away from subgrouping “to stop trying to ‘carve meatloaf at the joints’ and instead recognize the essential shared features of the autism spectrum while attempting to individualize diagnosis through dimensional descriptors" (p. 541) [32]. While it is likely that the reliability of diagnosis will improve by using the broader ASD label (compared to using DSM-IV subtypes), to understand the biology of “the autisms," it is necessary to clarify not just the similarities but also the differences among subgroups.

## Subgrouping and the Use of “Specifiers"

DSM-5 holds back from listing subgroups by recommending the use of “specifiers" to record the severity of cardinal symptoms, current language and intellectual ability, onset age and pattern, and concurrent genetic/medical or environmental/acquired conditions [33]. The use of specifiers is likely to be a valuable addition. However, there is a need to grasp the nettle to provide a more fine-grained taxonomy for research and clinical purposes (e.g., for access to appropriate individualized services). We therefore suggest expanding the list of specifiers toward the identification of clear subgroups. Table 1 summarizes our preliminary expanded but nonexhaustive list of specifiers, discussed here:

**Table 1:** A preliminary expanded (but nonexhaustive) list of specifiers, toward the identification of subgroups.

Category	Specifier	Example
*Developmental pattern*	Pattern of atypical development	1. Age and pattern of onset/regression
		2. Trajectory of development
		3. Language onset
		4. Hyperlexia
*Sex/gender*	Biological sex	Male/female
	Sex/gender-adjusted autistic features	Statistical characterization of autistic trait (e.g., percentile) relative to sex/gender-specific norms
*Clinical phenotype*	Co-occurring condition	1. Epilepsy
		2. Macrocephaly
		3. Gastrointestinal disorders
		4. Immune disorders
		5. Hyperserotonemia
		6. Attention deficit/hyperactivity disorder
		7. Anxiety disorders
		8. Depressive disorders
		9. Tics/Gilles de la Tourette syndrome
		10. Obsessive-compulsive disorder
		11. Schizophrenia spectrum
		12. Dyslexia
		13. Personality disorders
		14. Self-injurious behaviors
		15. Sleep disruption
		16. Eating disorders
		17. Gender dysphoria
	Taxonomic formulation	1. Asperger syndrome
		2. “Aloof"/“passive"/“active but odd"/“loners" groups
	Motor abnormality	1. Types of motor stereotypy
		2. Coordination disorder
		3. Dyspraxia
*Cognitive profile*	Intelligence	1. IQ profile (including discrepancy among subtests)
		2. Savant memory
		3. Savant spatial skills
	Current language (structural properties)	1. Phonological/phonetic processing (including articulation)
		2. Prosodic processing
		3. Morphological processing
		4. Syntactic processing
		5. Semantic processing
		6. Receptive vs. expressive abilities
	Social cognition	1. Emotion perception and understanding
		2. Face recognition
		3. Emotional contagion
		4. Social orienting
		5. Social and nonsocial reward processing
		6. Affective empathy
		7. Sympathy
		8. Joint attention
		9. Pretend play
		10. Theory of mind/mental perspective taking
		11. Self-referential cognition
		12. Alexithymia
		13. Metacognitive awareness
	Executive function	1. Cognitive flexibility
		2. Planning
		3. Inhibitory control
		4. Attention shifting
		5. Working memory
		6. Time perception
	Bottom-up perceptual processing	1. Global-local perceptual processing
		2. Low-level perceptual function and discrimination
		3. Synesthesia
	Top-down information processing	1. “Central coherence" (global-local contextual processing)
		2. “Systemizing" (drive to construct rule-based systems, ability to understand rule-based systems, knowledge of factual systems)
*Genetics*	Syndromic autism	1. Fragile X syndrome
		2. Rett syndrome
		3. Tuberous sclerosis complex
		4. Timothy syndrome
		5. Down syndrome
		6. Phenylketonuria
		7. CHARGE syndrome
		8. Angelman syndrome
		9. PTEN macrocephaly syndrome
		10. Joubert syndrome
		11. Landau-Kleffner syndrome
		12. Prader-Willi syndrome
		13. Smith-Lemli-Opitz syndrome
		14. Neurofibromatosis
	Familial aggregation	Simplex vs. multiplex
	Gene-level variations	e.g., *ASTN2, AVPR1A, CACNA1C, CACNA1G, CDH8, CDH9, CDH10, CNTN4, CNTNAP2, DISC1, DPP6, DPYD, EN2 (Engrailed 2), FMR1, FOXP2, GABRA4, GABRB3, GluR6, GRIK2, GSTP1, HOXA1, HOXB1, ITGB3, MACROD2, MADCAM1, MAPK3, MBD5, MECP2, MET, NLGN3, NLGN4X, NRXN1, NRXN3, OXTR, PRKCB1, PRL, PRLR, PTCHD1/PTCHD1AS, PTEN, RELN (Reelin), SEMA5A, SERT (SLC6A4), SHANK1, SHANK2, SHANK3 (ProSAP2), SLC25A12, TSC1, TSC2, UBE3A*
	Copy number variations (CNVs)	(specify known ASD-association status, genetic loci, and deletion/duplication)
*Environmental risks*	Social deprivation	Early social isolation or neglect*
		*(specify timing: postnatal months X to Y)
	Environmental risk factor exposure	1. Rubella virus infection during gestation*
		2. Valproic acid exposure during gestation*
		3. Antidepressant exposure during gestation*
		*(specify timing: gestational weeks X to Y)

- *Developmental pattern*: Age and pattern of onset of atypical development should be recorded. This includes not only “regression" [34] but also language onset/development and atypical social, emotional, communicative, physical and general intellectual development. These developmental patterns may have etiological implications. Differences in the trajectories of changes in autistic features [35],[36] may also have etiological implications, and are relevant to clinical management and prognosis. Development of prognostic biomarkers (e.g., of language outcome) may be particularly useful.
- *Sex/gender*: There are substantial sex/gender-specific effects at a variety of levels (e.g., behavior and cognition [37],[38], genetics [39],[40], proteomics [41], and neuroanatomy [42],[43]), which contribute to heterogeneity. For this reason, sex/gender should not just be viewed as a demographic descriptor but also an important specifier toward subgrouping “the autisms." In the general population there is a sex/gender difference in the distribution of autistic traits [26],[44]. If we view autism as the extreme of a continuous distribution of autistic traits in the population, one important specifier could be a statistical measure of where an individual lies on a sex/gender-specific distribution (e.g., percentile or z-score). Any statistical characterization would require sex/gender-specific norms and thresholds for defining ASD. There are many precedents for sex/gender-specific statistical characterization in other fields of medicine. For example, “failure to thrive" is defined using sex-specific growth curves for infants, and anemia is defined using sex-specific norms of serum hemoglobin levels [45]. Currently, DSM-5 is sex/gender-blind: it uses identical diagnostic criteria for ASD for both males and females. Although it remains an open question whether sex/gender-specific norms, thresholds, or criteria should be adopted, we underscore the importance of sex/gender to aid future research into subgrouping, which may lead to the identification of important sex/gender-linked mechanisms [46]. This issue is especially relevant for understanding the male bias in prevalence and potential underrecognition of females [37],[44],[47].
- *Clinical phenotypes*: DSM-5 recognizes concurrent medical and neuropsychiatric conditions, which of course is vital as a substantial portion of individuals with ASD show comorbidity. In addition to these, we suggest ASD could also be specified by other *prototypical* clinical subgroups, such as *Asperger syndrome* (e.g., defined by Hans Asperger's initial report or the Gillberg criteria [48]) or Wing's categorization (of the “aloof," “passive," “active but odd," and “loners" groups) [49]. This would allow for more systematic investigation of these long-standing rich clinical descriptions, which have not been studied thoroughly yet. It is of particular concern that Asperger syndrome is not specified by DSM-5 given insufficient research being conducted into how this may differ from other forms of autism [50]. In the context of a relative lack of consensus in definition [51], research findings of similarities and differences between “Asperger syndrome" and so-called “high-functioning autism (autistic disorder)" can either be interpreted as not supporting such a distinction [52] or that it is premature to rule out the separateness of the two [53]. Providing that the definition of the diagnosis adopted in the study is well described, in research using large samples it should be straightforward to identify a reliable signal, and the concern of unsatisfactory interrater reliability [32] may be alleviated.
- *Cognitive profile*: Cognition plays a pivotal role linking brain and behavior in ASD [54], yet curiously remains absent in DSM-5. Cognitive specifiers would be relevant for research into subgrouping, for discovering biomarkers, and for clinical evaluation. While the variability of intelligence and structural properties of language (as well as their development) has long been acknowledged, and is predictive of prognosis [55],[56], other aspects of cognition in autism may also be useful. Examples include *social cognition* (e.g., mentalizing/theory of mind, emotion processing, social orienting and reward processing), *executive function* (e.g., cognitive flexibility, planning, inhibitory control, attention shifting), *bottom-up perceptual processing* (e.g., global-local perceptual processing, low-level perceptual function and discrimination), and *top-down information processing* (e.g., “central coherence," “systemizing"—the drive to analyze and construct rule-based systems). Although some of the measures for these cognitive domains still lack general population norms, this should not prevent researchers and clinicians from including a systematic cognitive assessment focusing on these domains, for both research into individual differences and for individualized service planning.
- *Known genetic correlates*: These have the potential to dissect “the autisms" into subgroups at the genetic level [57], so they are vital to record in both research and clinical settings [58]. This includes identifiable ASD-related genetic syndromes, chromosomal anomalies, and rare and highly penetrant de novo and non–de novo genetic variations (copy number variations and rare genetic variants) [57],[59]–[61]. Depending on the pattern of familial aggregation and the nature of genetic variation (de novo vs. non–de novo), autism may be further classified into “simplex" and “mutliplex." In addition, common inherited variations (i.e., polymorphisms) and de novo genetic variation of low penetrance in multiple genes in critical biochemical and cellular pathways associated with neurodevelopment [62] may underlie the autistic spectrum that extends on to the general population. Some of the candidate genes are listed in Table 1, but we refer the readers to https://gene.sfari.org/autdb/Welcome.do for a constantly updating database.
- *Potential environmental contributors*: Perhaps due to the frequently reported high heritability of autism [63], environmental factors and gene-environment interplay in autism are understudied [64] but may also help toward identification of subgroups. For example, social deprivation early in life [65] or other environmental risk factor exposure (e.g., teratogens) and the timing of exposure [66] could be relevant and should be specified.

It is notable that the US National Institute of Mental Health (NIMH) has initiated the Research Domain Criteria [67] (with many of the above domains covered) to provide a system independent of DSM and ICD. Such initiatives are important to move toward identification of subgroups in a research context and for how they cut across DSM/ICD diagnostic categories. Such a system could provide a basis for identifying biological mechanisms that may or may not respect the DSM/ICD classification boundaries.

## Conclusions

DSM-5 ASD criteria should be commended for its clearer symptom descriptions and grouping, for acknowledging the spectrum nature of autism, and for recognizing the dynamic nature of development and how individuals interact with their environment. Moreover, for clinical purposes a unitary label of ASD may be beneficial in planning the support systems for *all* individuals “on the spectrum" who require help from education and health- and social-care systems. However, it is important to remember that autism is not homogenous, and defining it using the umbrella term ASD risks whitewashing the evident heterogeneity, which has a substantial impact for research into this condition. The identification of core features of autism using the broader ASD label cannot overcome the existence of heterogeneity. It has simply moved us from the level of subgroups (“apples and oranges") to the prototypical level (“fruit"). We argue that to make progress in autism research, and ultimately to improve clinical practice, we need to move forward in the identification of subgroups within the autism spectrum.

Toward this end, we have expanded the list of possible dimensional and categorical “specifiers" to improve our recognition of “the autisms." In addition, it is important to clarify the different definitions of the term “spectrum." Given that the spectrum extends into the general population, research needs to address the relationship between cardinal autistic symptoms and associated autistic traits (such as excellent attention to detail). Finally, we need to be fully aware of the inherent limitations of the existing psychiatric diagnostic systems, and consider other approaches that may be beneficial for research purposes [68].

The practical implication of the arguments proposed in this article is that *parallel behavioral characterization systems* may be necessary for autism research from now on. Although DSM-5-based diagnoses are expected to be widely accepted by researchers, a list of specifiers (including and beyond those recommended here) to aid phenotypic characterization will prevent us from losing sight of “the autisms." In a world that is moving toward individualized medicine, not incorporating information about such specifiers will be a backward step. Last but not least, in order to provide the basis to compare with the rich studies to date and to accurately assess the impact of shifting from DSM-IV to DSM-5, a parallel record of DSM-IV diagnoses may be helpful for both research and clinical settings in which these issues are particularly of concern.

## Abbreviations

AQ: Autism Spectrum Quotient
ASC: Autism Spectrum Conditions
ASD: Autism Spectrum Disorder
ASSQ: Autism Spectrum Screening Questionnaire
BAP: broader autism phenotype
CAST: Childhood Autism Spectrum Test
DSM: Diagnostic and Statistical Manual of Mental Disorders
ICD: International Classification of Diseases
NIMH: US National Institute of Mental Health
PDD: Pervasive Developmental Disorder
Q-CHAT: Quantitative Checklist for Autism in Toddlers
SRS: Social Responsiveness Scale
